# Causes of death and post-mortem testing for SARS-CoV-2 in a tertiary hospital during the COVID-19 pandemic in Ghana

**DOI:** 10.4102/ajlm.v11i1.1766

**Published:** 2022-11-23

**Authors:** Edward Asumanu, Seth Attoh, Raymond X. Servor, Clement Laryea, Mary McAddy, Fred Hobenu, Raymond Factchu, Kwesi Agyemang-Bediako, Edward O. Nyarko, Godwin K. Nyarko, Marcus K. Moroti, Lawrence Edusei

**Affiliations:** 1 Postgraduate College, 37 Military Hospital, Accra, Ghana; 2 Department of Anatomical Pathology, 37 Military Hospital, Accra, Ghana; 3 Department of Medicine, 37 Military Hospital, Accra, Ghana; 4 Public Health Division, 37 Military Hospital, Accra, Ghana

**Keywords:** post-mortem, severe acute respiratory syndrome coronavirus 2, coronavirus disease 2019, surveillance, pathology

## Abstract

**Background:**

Causes of death during the coronavirus disease 2019 (COVID-19) pandemic ranhttp://crossmark.crossref.org/dialog/?doi=10.4102/ajlm.v11i1.1766=pdf&date_stamp=2022-11-23ge from direct consequences of severe acute respiratory syndrome coronavirus 2 (SARS-CoV-2) infection to deaths unrelated to SARS-CoV-2. Another feature of the pandemic is the post-mortem testing for SARS-CoV-2. Understanding these aspects of COVID-19 are essential in planning and limiting the impact of SARS-CoV-2 virus on healthcare systems.

**Objective:**

This study investigated the underlying causes of death and the presence of SARS-CoV-2 in bodies received at the 37 Military Hospital, Accra, Ghana, during the COVID-19 pandemic.

**Methods:**

The study was conducted from 4–27 May 2020. Deceased patients that met the inclusion criteria were prospectively selected during the expanded surveillance period for SARS-CoV-2 testing, autopsy and determination of underlying and immediate cause of death.

**Results:**

A total of 161 deceased patients were analysed with 53 autopsies. The overall positive test rate for SARS-CoV-2 was 14.9% (24/161 patients), with a positive rate of 5.0% (8/161 patients) for nasopharyngeal samples and 30.2% (16/161 patients) for bronchopulmonary samples. The underlying causes of death were not related to SARS-CoV-2 infection in 85.1% (137/161) of patients, SARS-CoV-2-associated 12.4% (20/161) and SARS-CoV-2-induced in 2.5% (4/161). Cardiovascular complications formed the most common cause of death in patients with or without SARS-CoV-2.

**Conclusion:**

There was a high positive rate of SARS-CoV-2 in post-mortem cases. However, most deaths were not caused by SARS-CoV-2 but by cardiovascular complications. The high rate of bronchopulmonary positive results for SARS-CoV-2 requires that autopsies be done in suspicious cases with negative nasopharyngeal sampling.

## Introduction

In 2019, the world was struck by the severe acute respiratory syndrome coronavirus 2 (SARS-CoV-2) which caused the coronavirus disease 2019 (COVID-19).^1,2^ The resulting COVID-19 pandemic continues to pose challenges to healthcare systems. Compared to previous pandemics, the COVID-19 pandemic has exhibited a cyclical nature and unique manifestation in different geographical areas.^2^

Various aspects of the disease have been reported from epidemiology to mortality.^3,4,5,6,7^ Studies on mortality do not report on other causes of death within the pandemic,^1,6,7^ although these are equally important in the fight against the SARS-CoV-2. Autopsies are indispensable in clarifying the cause of death, as well as for quality control and audit in medical practice. It is an established fact that a clinically determined cause of death is not as reliable as an autopsy-determined cause of death.^8,9^

Real-time reverse transcription polymerase chain reaction (rRT-PCR) is the standard laboratory technique for testing for SARS-CoV-2 and can be used for samples taken at autopsy.^10^ Identifying pathological consequence of SARS-CoV-2 at autopsy can be a useful addition to rRT-PCR.

Surveillance has remained a cornerstone in the management of the COVID-19 pandemic. Different types of surveillance systems have been used during the COVID-19 pandemic, such as the hospital-based mortality surveillance and routine surveillance.^11^ As part of its expanded surveillance protocol, the 37 Military Hospital in Accra, Ghana, screened all deceased patients received at the Department of Anatomical Pathology morgue for SARS-CoV-2 and cause of death in May 2020. The aim of the study was to determine the underlying causes of death and the presence of SARS-CoV-2 using rRT-PCR for all cases admitted to the Department of Anatomical Pathology during the study period.

## Methods

### Ethical considerations

Permission was granted by the 37 Military Hospital’s institutional review board for the study with ethical approval number 37MH-IRB IPN/MAST/2020. No consent was required of the next of kin for the autopsies. To ensure privacy and confidentiality of the deceased patients, data was de-identified, coded and stored with passwords on electronic media.

### Study design

This prospective study was conducted over a three-week period from 4 to 27 May 2020. This was the first institutional surveillance study in Ghana on deceased patients at an Anatomical Pathology department. The study was conducted at the J.M. Wadhwani Department of Anatomical Pathology at the 37 Military Hospital in Accra, Ghana. The department receives about 4000 deceased individuals of all categories of death from the hospital and its environs annually and conducts approximately 1500 autopsies. The department conducts autopsies for patients who have died from unknown cause for medico-legal or coroner’s inquiry, homicide, suicide, accidents, misadventure and routine hospital cases for autopsy (where the cause of death is not known by the attending physician). Deceased patients with a known cause of death, where a medical certificate of cause of death has been provided by the attending physician, are stored by the department prior to burial.^12^

Two categories of deceased patients, in-hospital deaths (IHDs) and brought-in-dead cases (BIDs), formed the study population. In-hospital deaths are deaths that occurred while patients were being managed in the hospital. Brought-in-dead deaths are those that occurred outside the hospital or before the individual had been seen by a physician in the hospital. All deceased individuals received 4–27 May 2020 were included in the study, except for individuals less than one year old, unidentified individuals and patients diagnosed as having COVID-19 in the hospital.

Study procedures were conducted in three phases. First, samples were collected for SARS-CoV-2 testing. Second, an autopsy was completed for a subset of patients when requested by the coroner or physician. Finally, the underlying and immediate cause of death for all individuals was determined.

### Sample collection for SARS-CoV-2

Nasopharyngeal samples were collected from all deceased patients who met the inclusion criteria by the anatomical pathology team using sterile swab sticks. Additional bronchopulmonary samples were collected from patients who underwent autopsy as part of their post-mortem examinations. For the bronchopulmonary specimens, one sample was taken from swabs of secretions in the tertiary bronchi and another from fluid squeezed from the lungs, after sectioning, into an ordinary sterile specimen container. These samples were sent for rRT-PCR test at a national reference laboratory, Noguchi Memorial Institute for Medical Research in Accra, Ghana.

### Autopsy

A complete autopsy was done by certified department pathologists on individuals only when directed by the coroner or referred by the attending physician as per department policy. All available case notes and records were reviewed by the pathologists. Selected organs, including the lungs, heart, kidney, liver, spleen, intestines and brain, were sampled for histopathological examination and some photographs taken.

### Determination of underlying cause of death

Cause of death was considered to be the disease or injury which initiated the train of events leading directly to death or the circumstances of an accident or violence which produced the fatal injury. At least two certified pathologists determined the underlying cause of death for all individuals studied and reported on the histological findings. The pathologists analysed the clinical notes, as well as the cause of death provided by the attending physician, to determine the underlying cause of death for cases that did not undergo autopsy. In cases that underwent autopsy, the underlying cause of death was determined by evidence after histopathological examination and using the clinical notes, if any.

### Data analysis

The data collection form captured the biodata and the clinical cause of death for all patients who did not undergo autopsy. The cause of death for those who underwent autopsy was also recorded. The rRT-PCR test results were received in batches from the Noguchi Memorial Institute for Medical Research and matched to the individual patient to complete the data collection process. Underlying causes of death were categorised into three groups. The first group comprised deaths not related to SARS-CoV-2 infection. This group included patients who tested negative for SARS-CoV-2 and who had various underlying causes of death (e.g. massive cerebral haemorrhage due to hypertension); these are referred to as ‘non-SARS-CoV-2-related deaths’. The second group comprised deaths associated with, but not caused directly by, SARS-CoV-2 infection. This group included patients with a positive SARS-CoV-2 test but whose underlying cause of death was not directly attributable to SARS-CoV-2 (e.g., perforated duodenal ulcer); these are referred to as ‘SARS-CoV-2-associated deaths’. The last group comprised of deaths directly induced by SARS-CoV-2 infection. This group included patients with a positive SARS-CoV-2 test and whose underlying cause of death was attributable to SARS-CoV-2 (e.g., acute respiratory distress syndrome with or without pneumonia); these are referred to as ‘SARS-CoV-2-induced deaths’. Collected data were managed in Microsoft Excel (2016; Norristown, Pennsylvania, United States), which was used to calculate frequencies and proportions.

## Results

### Study population

A total of 161 deceased patients, including 96 IHDs and 65 BIDs, were examined over the study period (Table 1). The mean age of the study population was 53.3 years (Range: 18 months – 90 years) with a male-to-female ratio of 1:1. Among the age groups above 40 years, there was an increasing ratio of SARS-CoV-2 infection compared to non-SARS-CoV-2 infection. A total of 53 autopsies were performed. All deceased patients had nasopharyngeal samples collected for testing, and those that underwent full autopsy (*n* = 53) also had a bronchopulmonary sample collected. There was a total of 24 rRT-PCR tests positive for SARS-CoV-2 from both nasopharyngeal and bronchopulmonary samples. The overall positive test rate from the study population was 14.9% (24/161), with a positive rate of 5.0% (8/161) for nasopharyngeal samples and 30.2% (16/53) bronchopulmonary samples. Of patients who had no autopsy, eight had SARS-CoV-2-associated causes of death and a positive nasopharyngeal test and 100 had a negative nasopharyngeal test and a cause of death that was not associated with SARS-CoV-2. Eight patients with a negative nasopharyngeal test had a bronchopulmonary specimen that tested positive for SARS-CoV-2. Of the 161 patients included in the study, four patients’ (2.5%) deaths were directly caused by SARS-CoV-2, 20 patients (12.4%) tested positive for SARS-CoV-2 but had another cause of death; for the majority of patients, the cause of death was not related to SARS-CoV-2 infection (*n* = 137, 85.1%).

**TABLE 1:** Characteristics of deceased individuals examined for SARS-CoV-2 infection, 37 Military Hospital, Accra, Ghana, 4–27 May 2020.

Characteristic	All individuals		SARS-CoV-2 positive†		SARS-CoV-2 negative†	
	No.	Percentage	No.	Percentage	No.	Percentage
All individuals	161	-	24	14.9	137	85.1
**Gender**						
Male	81	50.3	10	12.3	71	87.7
Female	80	49.7	14	17.5	66	82.5
**Age (mean: 53.3 years)**						
1–20	13	8.1	1	7.7	12	92.3
21–40	26	16.2	4	15.4	22	84.6
41–60	63	39.1	8	12.7	55	87.3
61–80	44	27.3	8	18.2	36	81.8
81 or older	15	9.3	3	20.0	12	80.0
**Deceased individuals examined**						
In-hospital death	96	59.6	12	7.6	115	71.4
Brought-in-dead	65	40.4	12	7.6	75	46.6
**Autopsy** ‡						
No	108	67.1	8	5.1	100	62.1
Yes	53	32.9	16	10.0	37	23.0
**Sample type**						
Nasopharyngeal	161	100	8	5.0	153	95.0
Bronchopulmonary§	53	32.9	16¶	30.2	37	23.0
**Cause of death**						
Non-SARS-CoV-2 related	137	85.1	-	-	137	85.1
SARS-CoV-2 associated	20	12.4	20	12.4	-	-
SARS-CoV-2 induced	4	2.5	4	2.4	-	-

SARS-CoV-2, severe acute respiratory syndrome coronavirus 2; No., number.

† , Real-time reverse transcription polymerase chain reaction assay;

‡ , Autopsy was conducted only upon request from coroner or attending physician;

§ , Bronchopulmonary samples were collected only from individuals who underwent autopsy;

¶ , Eight patients’ nasopharyngeal tests were negative.

### SARS-CoV-2-induced deaths

**FIGURE 1:**
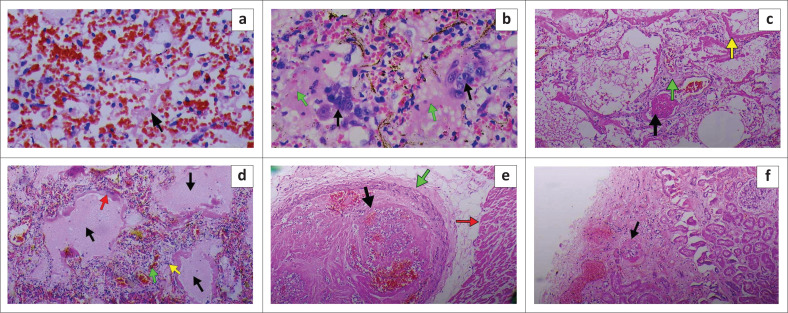
Haematoxylin and eosin staining of various organs in SARS-CoV-2-induced deaths at the 37 Military Hospital, Accra, Ghana, May 2020. (a) Severe congestion with intra-alveolar deposition of fibrinous tissue (black arrow) ×400. (b) Type 2 pneumocyte hyperplasia (black arrows), diffuse alveolar damage (DAD) with early hyaline membrane (HM) formation (green arrows) ×400. (c) DAD with prominent HM formation (yellow arrow), interstitial fibrosis (green arrow) and a thrombus in a small pulmonary artery (black arrow) ×100. (d) DAD with prominent HM (red arrow), oedema (black arrows), congestion (green arrow), interstitial fibrosis and lymphocytic infiltrates (yellow arrow) ×100. (e) Thrombus formation (black arrow) in a branch of a coronary artery (green arrow) in the heart muscle (red arrow) ×400. (f) Micro-thrombus (black arrow) in a renal arteriole ×100.

Of the four SARS-CoV-2-induced deaths, three were IHDs and one was BID (Table 2). All cases had severe pulmonary oedema and diffuse alveolar damage (DAD) with prominent hyaline membrane formation (Figure 1). There was severe congestion with intra-alveolar deposition of fibrinous tissue in Case 1 (Figure 1a). Case 2 also showed large pneumocytes with enlarged nuclei and granular amphophilic cytoplasm and microthrombi in the smaller pulmonary arteries (Figures 1a and Figure 1c) as well as edema, congestion, proliferation of fibroblasts and lymphocytic infiltrates in the interstitium (Figure 1d). Case 3 showed very prominent DAD and hyaline membrane formation, interstitial fibrosis, infiltration of the pulmonary interstitium and alveolar spaces by macrophages and scattered multinucleated giant cells. Microthrombi were also noted in smaller pulmonary arteries (Figure 1c). Case 4 revealed thrombi in branches of the coronary and renal arteries (Figure 1e and Figure 1f).

**TABLE 2:** Summary of SARS-CoV-2 induced deaths during post-mortem at the 37 Military Hospital, Accra, Ghana, May 2020.

Case no.	Age	Gender	Category	Comorbidities	Nasopharyngeal specimen	Bronchial specimen
1	42	M	BID	Nil	Positive	Positive
2	55	M	IHD	Hypertension diabetes mellitus	Positive	Positive
3	69	F	IHD	Hypertension heart failure	Positive	Positive
4	39	F	IHD	HIV infection	Negative	Positive

IHD, in-hospital deaths; BID, brought-in-dead deaths; no., number; F, female; M, male.

### SARS-CoV-2-associated deaths

Of the 20 patients with a positive SARS-CoV-2 test and an unrelated cause of death, 13 were IHD and seven were BID (Table 3). The mean age was 54.7 with a range of 24–90 years. The male:female ratio was 1:1. Cardiovascular disorders constituted almost half of the underlying causes of death in this category (*n* = 9). Hypertension was the most common cardiovascular disorder among both IHDs and BIDs. Other causes of death included tumours (*n* = 1), accidents and trauma (*n* = 4), infections (*n* = 1), and others (*n* = 5).

**TABLE 3:** Summary of SARS-CoV-2 associated deaths during post-mortem at the 37 Military Hospital, Accra, Ghana, May 2020.

Underlying cause of death	Immediate cause of death	IHD	BID	Total
All SARS-CoV-2-associated deaths		13	7	20
**Cardiovascular disorders**				
All cardiovascular disorder-related causes	-	5	4	9
Hypertension	Haemorrhagic stroke	2	1	3
	Heart failure	1	-	1
	Sudden cardiac death	1	1	2
Atherosclerosis	Cerebral infarct	1	1	2
Ischaemic heart disease	Ischaemic heart disease	-	1	1
**Tumours**				
Benign prostatic hyperplasia	Bleeding benign prostatic hyperplasia	1	-	1
**Accident and trauma**				
All accident and trauma-related causes	-	4	4	8
Road traffic accident	Severe head injury	3	-	3
	Perforated duodenal ulcer	1	-	1
**Infections**				
Pneumonia	Septicaemia	1	-	1
**Others**				
All other causes	-	2	3	-
Deep vein thrombosis	Pulmonary embolism	1	2	3
Gastrointestinal bleeding	Haemorrhagic shock	1	-	1
Alcoholic hepatitis	Liver failure	-	1	1

**Total**	**-**	**13**	**7**	**20**

IHD, in-hospital deaths; BID, brought-in-dead deaths; SARS-CoV-2, severe acute respiratory syndrome coronavirus 2.

### Non-SARS-CoV-2-related deaths

Of the 137 non-SARS-CoV-2-related deaths, 80 were IHD and 57 were BID. The age range was 2–88 years with a male: female ratio of 1:1 For patients who had an autopsy, the clinical diagnoses were confirmed, except for one case of primary liver cell carcinoma, which had been diagnosed clinically as cirrhosis of the liver. Broad categories of causes of death identified were: cardiovascular disorders (*n* = 49), tumours (*n* = 23), accident and trauma (*n* = 13), diabetes mellitus-related complications (*n* = 11), infections (*n* = 18), liver disorders (*n* = 6), renal disorders (*n* = 1), and others (*n* = 16). Hypertension and its related complications formed the most common cause of death in patients without SARS-CoV-2 (Table 4). Two patients who tested negative for SARS-CoV-2 on rRT-PCR for both the nasopharyngeal and bronchoalveolar samples had lung tissue histopathology consistent with acute respiratory distress syndrome or DAD. There were five IHD and two BID with ‘miscellaneous’ causes of death. The BID deaths were from viral hepatitis (*n* = 1) and severe asthma (*n* = 1), whereas the IHD deaths were from intravascular haemolysis (*n* = 1), viral meningitis (*n* = 1) and malaria (*n* = 3).

**TABLE 4:** Summary of non-SARS-CoV-2 related deaths at the 37 Military Hospital, Accra, Ghana, May 2020.

Underlying cause of death	Immediate cause of death	IHD	BID	Total
All	-	96	65	-
**Cardiovascular disorder**				
All cardiovascular disorders	-	29	20	49
Hypertension	Haemorrhagic stroke	6	3	9
	Heart failure	7	1	8
	Hypertensive heart disease	-	4	4
Cerebral atherosclerosis	Cerebral infarct	9	3	12
Ischaemic heart disease	Acute myocardial infarction	1	5	6
	Heart failure	2	3	5
Valvular heart disease	Heart failure	2	-	2
Dilated cardiomyopathy	Heart failure	1	1	2
Berry aneurysm	Ruptured aneurysm	1	-	1
**Tumours/tumour-like**				
All tumours/tumour-like causes	-	11	8	19
Prostate cancer	Metastatic prostate cancer	4	2	6
Breast cancer	Metastatic breast cancer	2	1	3
Pancreatic cancer	Liver failure	1	-	1
Stomach cancer	Severe anaemia	2	-	2
Cervical cancer	Metastatic cervical cancer	1	-	1
Ovarian sarcoma	Metastatic ovarian sarcoma	1	-	1
Lung cancer	Lung cancer	-	1	1
Chronic myeloid leukemia	Severe anaemia	1	-	1
Brain tumour	Raised intracranial pressure	1	1	2
Benign prostatic hyperplasia	Bleeding benign prostatic hyperplasia	1	-	1
	Acute pyelonephritis	1	1	2
Uterine leiomyoma	Bleeding uterine fibroid	-	2	2
**Accident and trauma**				
All accident and trauma-related causes	-	10	3	13
Road traffic accident	Severe head injury	7	-	7
	Chest injuries	1	-	1
	Perforated duodenal ulcer	1	-	1
	Multiple soft tissue and bony injuries	1	-	1
Electrocution/Drowning	Asphyxia	-	3	3
**Diabetes mellitus**				
All diabetes mellitus-related causes	-	7	4	11
Diabetes mellitus	Myocardial infarction	-	1	1
	Severe diabetes mellitus	3	2	5
	Pyelonephritis	-	1	1
	Benign ulcers	2	-	2
	Pneumonia	1	-	1
	Fracture	1	-	1
**Infections**				
All infection-related causes	-	10	9	19
Pneumonia	Septicaemia	4	4	8
Infective gastroenteritis	Septicaemia	1	-	1
	Hypovolaemic shock	1	-	1
Cellulitis	Cellulitis	-	2	2
Retrovirus infection	Severe anaemia	2	1	3
	Lymphoma	-	1	1
	Tuberculosis	1	-	1
	Meningitis	1	-	1
**Renal disorders**				
Chronic glomerulonephritis	Chronic renal failure	-	1	1
**Liver disorders**				
All liver-disorder-related causes	-	3	6	9
Liver cirrhosis	Raptured hepatoma	1	2	3
	Liver failure	1	4	5
	Raptured oesophageal varices	1	-	1
**Others**				
All other causes	-	8	7	15
Deep vein thrombosis	Pulmonary embolism	2	4	6
Sickle cell disease	Acute chest syndrome	2	1	3
Miscellaneous	-	4	2	7

**Total**	**-**	**80**	**57**	**137**

IHD, in-hospital deaths; BID, brought-in-dead deaths.

## Discussion

The study identified three categories of deaths that occurred during the pandemic. There were a significant number of undiagnosed SARS-CoV-2 infections among deceased individuals received by the Anatomical Pathology Department of the 37 Military Hospital with unique histopathological features of SARS-CoV-2 induced deaths. The potential for community spread of the virus from handling of unsuspected SARS-CoV-2 deceased individuals without personal protective equipment, as well as the SARS-CoV-2 cases found during autopsy, are a missing link in efforts to control the pandemic. There are other documented missing links affecting overall SARS-CoV-2 control such as non-uniform data collection, vaccine hesitancy and non-reliable energy infrastructure in low-resource environments.^13,14,15^ In an Italian multicentre study conducted in 2020, the presence of SARS-CoV-2 in bronchoalveolar samples was 32.7% of suspected cases with 76% having a double negative nasopharyngeal test.^16^ Our study also found SARS-CoV-2 in the bronchopulmonary specimens of cases with negative nasopharyngeal tests and in cases who died from other conditions. These findings need to be factored into the current health policy on SARS-CoV-2.

The underlying cause of death is critical in accurate reporting and documentation for better epidemic surveillance and effective planning of control measures.^17^ This is the first reported post-mortem study data for Ghana that has categorised underlying causes of death into three groups: non-SARS-CoV-2 related (85.1%), SARS-CoV-2 associated (12.4%) and SARS-CoV-2 induced (2.5%). The case fatality rate in Ghana for SARS-CoV-2 infection was reported to be an average of 0.5% in July 2020.^18^ This did not include deaths due to SARS-CoV-2 as identified by post-mortem examinations. The presence of post-mortem-diagnosed cases of SARS-CoV-2-induced deaths in this study implies that the overall case count is higher than reported for Ghana. The need to include cases identified by pathology departments during post-mortem examinations in defining all case mortality has been emphasised.^19^ This highlights the importance of post-mortem examination in understanding the natural history of diseases and defining a true mortality rate.

Accurate reporting is a requirement in the documentation of cause of death. In its guidelines for certification and classification of COVID-19 as a cause of death, the WHO requires a distinction between underlying cause of death and immediate cause of death in the completion of Part I of the International Form of the Medical Certificate of Cause of Death.^20^ This was to encourage uniformity of data collection to guide health policy, but differences in classification are emerging.^20,21^ The evolution of the pandemic and the understanding of the disease requires a simple categorisation of underlying of causes of death. The categorisation adopted in this study is useful for determining accurate case counts during clinical audits of deaths.

Identifying subgroups of COVID-19 case mortality is useful in managing the pandemic.^22^ Of particular interest were the 20 SARS-CoV-2-associated deaths in our study. These cases were categorised as having died from their underlying medical conditions, rather than from SARS-CoV-2. The prioritisation of care for SARS-CoV-2 infections by the health system, rather than the underlying condition, might have contributed to their mortality. Most deaths during the pandemic have been reported as being related to underlying conditions.^8,23^ This raises the question as to whether SARS-CoV-2 has the potential to delay care among patients with underlying medical conditions. The presence of SARS-CoV-2 among the BIDs patients in our study suggests a high community transmission of the disease. Such cases may be asymptomatic and likely to result in an increased disease burden in the absence of strict protocols.

Interestingly, our study found a high proportion (85.1%) of deaths not attributable to SARS-CoV-2. The impact of the pandemic on health systems and the lack of provision of optimum care are attributable causes to the phenomenon of high proportions of non-SARS-CoV-2-related deaths.^24^ Among the BID cases in our study not attributable to SARS-CoV-2, hypertension, infection, liver cirrhosis, diabetes mellitus and pulmonary embolism-related complications were the leading causes of death. Causes of death not attributable to SARS-CoV-2 for cases from the hospitals (in descending order) included hypertension, tumours, accident and trauma, diabetes mellitus and infections. In Ghana, the prevalence of major non-communicable diseases, especially hypertension, and their risk factors has increased and contributes significantly to the disease burden.^25^ Hypertension and its related complications were the leading causes of death in both groups of patients. Non-communicable metabolic diseases, tumours and trauma-related deaths rank high in the study. This emphasises the need for aggressive clinical care for non-communicable diseases even in the face of a positive SARS-CoV-2 test.

Our study found an overall SARS-CoV-2 case positivity rate of 14.90% among deceased patients received by the Anatomic Pathology Department; these are not included in Ghana’s national data on SARS-CoV-2. The study found a mean age of 55 years for male patients and 53 years female patients with an equal gender ratio. This is a different demographic pattern from the reported increased susceptibility to COVID-19 among the elderly, those with severe comorbidities and among male patients.^26^ While our study found a high proportion of comorbidities among the elderly, the deaths were not directly from SARS-CoV-2, but from the associated cormorbidities.^23^ It can be extrapolated that, in the context of an underlying medical condition, the primary disease possess more risk than the SARS-CoV-2 infection.

Severe acute respiratory syndrome corona virus 2 has been known to survive longer in cold temperatures and on fomites, with guidelines emerging for the handling of an autopsy in suspected cases.^27,28^ In our study, the pathology department discovered 24 SARS-CoV-2-infected deceased patients, of which 20 were previously undiagnosed. The absence of appropriate personal protective equipment and standard protocols predisposed staff to a high risk of infection. It is recommended that there should be a national protocol for the safe performance of autopsies during the pandemic.

The site of sample collection has a direct correlation with the sensitivity of rRT-PCR tests, and bronchopulmonary samples give the highest viral yield.^29,30,31^ In our study, bronchopulmonary specimens gave a higher positivity rate than nasopharyngeal specimens, implying that our bronchopulmonary specimens also had a higher viral load. The higher level of virus in bronchial secretions should be a recognised risk for medical staff during oropharyngeal endoscopic procedures and anaesthesia. The necessary awareness and preventive practices to avoid contracting the virus should be in place.

The analysis of the histology of specimens from COVID-19 cases provides useful information about the disease. Our study found inflammatory features in the lungs and thrombus formation in the arterial systems of major organs. This combination of pulmonary congestion with peri-vascular lymphocytic infiltrates, interstitial/intra-alveolar fibrosis, type 2 pneumocyte hyperplasia, DAD with hyaline membrane formation, as well as arterial thrombosis, are likely to result in the rapid and severe hypoxemia noticed among COVID-19 patients. An ensuing cellular response in the presence of an underlying medical disease could contribute to the high mortality in these patients. A higher proportion of such patients in our study had cardiovascular disease. The role of the thrombus formation and subsequent hypoxia on vascular endothelium need to be investigated.

In our study, there were two cases with the clinical and histopathological features of acute respiratory distress syndrome/DAD that had negative SARS-CoV-2 rRT-PCR test results. There were no definitive diagnoses at the time of autopsy. Even though both patients had comorbid conditions, evidence at post-mortem did not attribute their deaths to the comorbid conditions. A recent case series report ascribed such situations to a ‘probable’ case of COVID-19 as the cause of death.^30^ Such cases, who test negative for SARS-CoV-2 but have clinical features suggestive of COVID-19, should be considered as a subgroup of COVID-19 to help in case management and prevention protocols, considering the high mortality associated with the disease. The negative PCR results of these two cases are most likely to be false-negative results. Such false-negative errors may be the result of pre-analytical issues,^32^ such as poorly collected samples, mislabelling, improper storage or transport, or interference from medications. That notwithstanding, there are other causes of acute respiratory distress syndrome/DAD that should be considered, and an autopsy should therefore be encouraged to distinguish between SARS-CoV-2-*induced* deaths and SARS-CoV-2-*associated* deaths.

The study showed that a significant number of clinically diagnosed cases of COVID-19 may be false. Deaths could have been wrongly labelled as due to COVID-19, because of a positive SARS-CoV-2 test by the nasopharyngeal route and presence of respiratory difficulty. Such patients would have been denied dignified burials by their relatives, instead of the practice of mass burial as mandated by national policy at the beginning of the pandemic. These findings are a useful addition to Ghana’s national data to determine which deceased patients can be safely released to families and which require supervised burials. The importance of routine screening in high-risk clinical practice among asymptomatic patients and guidelines to protect staff must be emphasised.

### Limitations

It would have been ideal to conduct autopsies for all 161 patients in this study, in order to determine the pathological changes present, presence of SARS-CoV-2 in bronchopulmonary specimens, as well as the autopsy cause of death. Unfortunately, this was not possible, because in some cases a medical certificate of cause of death had been issued by the attending physician so no autopsy was requested. This study was also limited to one institution and may not reflect the nationwide pattern.

### Conclusion

There was a high positive rate of SARS-CoV-2 infection among the patients in this study who underwent post-mortem examination. However, the majority of those deaths were not caused by SARS-CoV-2 but by cardiovascular complications. Thus, there should be aggressive clinical care for non-communicable diseases. The high SARS-CoV-2 infection rate among the deceased may reflect a high rate in the population. The high rate of bronchopulmonary positive results for SARS-CoV-2 requires that autopsies be conducted in suspicious cases with negative nasopharyngeal sample test results. False-negative rRT-PCR test results for nasopharyngeal samples were common enough that there is a need to intensify preventive protocols.

## References

[1] Rahimi Pordanjani S, Hasanpour A, Askarpour H, et al. Aspects of epidemiology, pathology, virology, immunology, transmission, prevention, prognosis, diagnosis, and treatment of COVID-19 pandemic: A narrative review. Int J Prev Med. 2021 May 15;12:38. 10.4103/ijpvm.IJPVM_469_20 34249287 PMC8218815

[2] Sohrabi C, Alsafi Z, O’Neill N, et al. World Health Organization declares global emergency: A review of the 2019 novel coronavirus (COVID-19). Int J Surg. 2020 May;76:71–76. 10.1016/j.ijsu.2020.02.034 32112977 PMC7105032

[3] Zhao Z, Li X, Liu F, Zhu G, Ma C, Wang L. Prediction of the COVID-19 spread in African countries and implications for prevention and control: A case study in South Africa, Egypt, Algeria, Nigeria, Senegal and Kenya. Sci Total Environ. 2020;729:138959. 10.1016/j.scitotenv.2020.138959 32375067 PMC7182531

[4] Wang HJ, Du SH, Yue X, Chen CX. Review and prospect of pathological features of corona virus disease. J Forensic Med. 2020;36(1):16–20. 10.12116/j.issn.1004-5619.2020.01.004 32198986

[5] Lai W, Xie C, Pan H, Fan M, Liu J. Computed tomography of the lungs in novel corona virus (COVID-19) infection. Pediatr Radiol. 2020;50(7):1016–1017. 10.1007/s00247-020-04664-7 32358678 PMC7195298

[6] Ma K, Chen T, Han MF, Guo W, Ning Q. [Management and clinical thinking of coronavirus disease 2019]. Zhonghua Gan Zang Bing Za Zhi. 2020 Mar 3;28(0):E002. 10.3760/cma.j.issn.1007-3418.2020.0002 32125126

[7] Ahn DG, Shin HJ, Kim MH, et al. Current status of epidemiology, diagnosis, therapeutics, and vaccines for novel coronavirus disease 2019 (COVID-19). J Microbiol Biotechnol. 2020 Mar 28;30(3):313–324. 10.4014/jmb.2003.03011 32238757 PMC9728410

[8] Schönamsgruber N, Schröder C, Edler C, Püschel K, Sperhake JP, Schröder AS. Quality of external post-mortem examination and quality of death certificates at the University Hospital in Hamburg. Rechtsmedizin. 2019;29(4):281–286. 10.1007/s00194-019-0323-5

[9] Ermenc B. Comparison of the clinical and post mortem diagnoses of the cause of death. Forensic Sci Int. 2000;114(2):117–119. 10.1016/S0379-0738(00)00329-7 10967252

[10] Barton LM, Duval EJ, Stroberg E, Ghosh S, Mukhopadhyay S. COVID-19 autopsies, Oklahoma, USA. Am J Clin Pathol. 2020 May 5;153(6):852. 10.1093/ajcp/aqaa062 32275742 PMC7184436

[11] Ibrahim NK. Epidemiologic surveillance for controlling covid-19 pandemic: Types, challenges and implications. J Infect Public Health. 2020;13(11):1630–1638. 10.1016/j.jiph.2020.07.019 32855090 PMC7441991

[12] Anim JT. Autopsy practice in Ghana – Reflections of a pathologist. GMJ. 2015;49(2):112–119. 10.4314/gmj.v49i2.9 PMC4549826 26339096

[13] Bauer DC, Metke-Jimenez A, Maurer-Stroh S, et al. Interoperable medical data: The missing link for understanding COVID-19. Transbound Emerg Dis. 2021;68(4):1753–1760. 10.1111/tbed.13892 33095970 PMC8359419

[14] Rinkoo AV, Songara D, Sharma A, et al. Reliable energy and responsive built environment: The missing links in COVID-19 response in resource-limited settings. Trop Med Health. 2020;48(1):67. 10.1186/s41182-020-00255-2 32831577 PMC7419725

[15] Zizzo J. The missing link in the covid-19 vaccine race. Hum Vaccin Immunother. 2021 May 4;17(5):1326–1328. 10.1080/21645515.2020.1831859 33079612 PMC8078724

[16] Patrucco F, Albera C, Bellocchia M, et al. SARS-CoV-2 detection on bronchoalveolar lavage: An Italian multicenter experience. Respiration. 2020;99(11):970–978. 10.1159/000511964 33075793 PMC7649696

[17] Veeranna CH, Rani S. Cause of death certification in COVID-19 deaths. Indian J Crit Care Med. 2020;24(9):863–867. 10.5005/jp-journals-10071-23561 33132574 PMC7584845

[18] Sibiri H, Prah D, Zankawah SM. Containing the impact of COVID-19: Review of Ghana’s response approach. Health Policy Technol. 2021;10(1):13–15. 10.1016/j.hlpt.2020.10.015 33169101 PMC7641588

[19] Piccininni M, Rohmann JL, Foresti L, Lurani C, Kurth T. Use of all cause mortality to quantify the consequences of covid-19 in Nembro, Lombardy: Descriptive study. BMJ. 2020;369:m1835. 10.1136/bmj.m1835 32409488 PMC7223479

[20] Singh B. International comparisons of COVID-19 deaths in the presence of comorbidities require uniform mortality coding guidelines. Int J Epidemiol. 2021;50(2):373–377. 10.1093/ije/dyaa276 33432354 PMC7928837

[21] World Health Organization. Medical certification of cause of death: Instructions for physicians on use of international form of medical certificate of cause of death [homepage on the Internet]. 4th ed. World Health Organization; 1979 [cited 2021 Aug 03]. Available from: https://apps.who.int/iris/handle/10665/40557 PMC2916762 14916317

[22] Morfeld P, Erren TC. [Deaths in nine regions of Italy in February/March 2020: ‘Mortality excess loupe’ for SARS-CoV-2/COVID-19-epidemiology in Germany]. Gesundheitswesen. 2020 May;82(5):400–406. 10.1055/a-1160-5859 32356298 PMC7295299

[23] Attoh S, Segborwotso RP, Akoriyea SK, et al. COVID-19 autopsy reports from the Ga-East municipal and the 37 Military Hospitals in Accra, Ghana. Ghana Med J. 2020 Dec;54(4 Suppl):52–61. 10.4314/gmj.v54i4s.9 33976442 PMC8087362

[24] Abdullah ASM, Tomlinson B, Thomas GN, et al. Impacts of SARS on health care systems and strategies for combating future outbreaks of emerging infectious diseases. In: Knobler S, Mahmoud A, Lemon S, et al., editors. Learning from SARS: Preparing for the next disease outbreak: Workshop summary [homepage on the Internet]. Washington, DC: National Academies Press (US); 2004 [cited 2021 Aug 03]. Available from: https://www.ncbi.nlm.nih.gov/books/NBK92485/22553895

[25] De-Graft Aikins A, Addo J, Ofei F, Bosu W, Agyemang C. Ghana’s burden of chronic non-communicable diseases: Future directions in research, practice and policy. Ghana Med J. 2012;46(2 Suppl):1–3. 23661810 PMC3645141

[26] Jin JM, Bai P, He W, et al. Gender differences in patients with COVID-19: Focus on severity and mortality. Front Public Health. 2020 Apr 29;8:152. 10.3389/fpubh.2020.00152 32411652 PMC7201103

[27] Xue Y, Lai L, Liu C, Niu Y, Zhao J. Perspectives on the death investigation during the COVID-19 pandemic. Forensic Sci Int Synerg. 2020 Apr 9;2:126–128. 10.1016/j.fsisyn.2020.04.001 32412012 PMC7144508

[28] Santurro A, Scopetti M, D’Errico S, Fineschi V. A technical report from the Italian SARS-CoV-2 outbreak. Postmortem sampling and autopsy investigation in cases of suspected or probable COVID-19. Forensic Sci Med Pathol. 2020 Sep;16(3):471–476. 10.1007/s12024-020-00258-9 32399755 PMC7216855

[29] Wang X, Tan L, Wang X, et al. Comparison of nasopharyngeal and oropharyngeal swabs for SARS-CoV-2 detection in 353 patients received tests with both specimens simultaneously. Int J Infect Dis. 2020 May;94:107–109. 10.1016/j.ijid.2020.04.023 32315809 PMC7166099

[30] Attoh SA, Hobenu F, Edusei L, et al. Postmortem diagnosis of COVID-19: Antemortem challenges of three cases at the 37 Military Hospital, Accra, Ghana. Afr J Lab Med. 2020;9(1):1290. 10.4102/ajlm.v9i1.1290 PMC7670026 33235831

[31] Edler C, Schröder AS, Aepfelbacher M, et al. Dying with SARS-CoV-2 infection – An autopsy study of the first consecutive 80 cases in Hamburg, Germany. Int J Legal Med. 2020 Jul;134(4):1275–1284. 10.1007/s00414-020-02317-w 32500199 PMC7271136

[32] Payne D, Newton D, Evans P, et al. Preanalytical issues affecting the diagnosis of COVID-19. J Clin Pathol. 2021;74:207–208. 10.1136/jclinpath-2020-206751 32631944

